# Role and Function of Frontline Health Workers During the COVID-19 Pandemic in a Rural Health Center in Kerala: A Qualitative Study

**DOI:** 10.7759/cureus.69128

**Published:** 2024-09-10

**Authors:** Hitha V Nair, Navami Sasidharan, Aswathy Sreedevi, Rahul U Ramachandran

**Affiliations:** 1 Community Medicine, Amrita Institute of Medical Sciences, Kochi, IND; 2 Child Health, National Health Mission, Government of Kerala, Thiruvananthapuram, IND

**Keywords:** accredited social health activist, barriers, challenges, covid 19, pandemic

## Abstract

Introduction

Accredited Social Health Activists (ASHAs) are community health workers established under the National Rural Health Mission (NRHM). ASHAs have played a crucial role in responding to the COVID-19 pandemic. The COVID-19 pandemic has prompted ASHAs to adapt their strategies and methods to effectively address the challenges and ensure the well-being of the communities they serve.

Objectives

This study was done to explore the impact of the COVID-19 pandemic on the duties and performance of ASHAs.

Methods

A qualitative study was conducted over one month in the rural Primary Health Center (PHC) area of Thrissur district, Kerala. Focus group discussions (FGDs) were conducted among ASHAs and in-depth interviews with a Junior Health Inspector (JHI) and a Junior Public Health Nurse (JPHN).

Results

During the COVID-19 pandemic, the healthcare system shifted its focus toward managing the virus, leading to changes in the responsibilities of ASHA workers. They were assigned additional duties, and their routine services were disrupted due to lockdown measures. Despite the challenges, several factors played a crucial role in facilitating their services, including social support, teamwork, and personal satisfaction. However, there were several barriers that affected their effectiveness, such as inadequate and inconsistent remuneration, lack of recognition, transportation difficulties, safety concerns, and out-of-pocket expenses.

Conclusion

ASHAs effectively managed the challenging situation during the COVID-19 pandemic by taking diligent measures and showing a high level of commitment to their duties. It is essential to prioritize regular evaluations of their performance to ensure ongoing quality and effectiveness.

## Introduction

Accredited Social Health Activists (ASHAs) are community health workers established under the National Rural Health Mission (NRHM). Their primary role is to raise awareness in the community about various health-related aspects and their determinants. ASHAs actively engage in local health planning, contributing to improved utilization of healthcare services within the community. They are the promoters of good health practices and will also provide a minimum package of curative care as appropriate and feasible for their level and make timely referrals. Their active involvement plays a vital role in promoting healthcare accessibility and addressing the specific needs of the local population [1].

There are approximately 985,690 ASHA workers deployed across India, with many of them (919,231) serving in rural areas. Kerala has a total of 24,513 ASHA workers, 22,117 of whom are in rural areas and 2,396 in urban areas [2]. The general norm for ASHA worker distribution is one ASHA per 1,000 population [3].

The COVID-19 pandemic had a profound impact on the work patterns and priorities of ASHA workers. ASHA workers took up new responsibilities during the COVID-19 pandemic, including raising awareness, conducting screening for vulnerable populations, facilitating referrals, delivering care to COVID-19 patients, and offering support to their families [4].

With the start of the COVID-19 pandemic, the role of ASHA workers has assumed tremendous importance as the governments depended heavily on them for community-level efforts to contain the outbreak [5]. Execution of these tasks resulted in an increased burden and health hazards for ASHAs and their relatives, resulting in heightened levels of anxiety and strain. Despite these challenges, they have managed to fulfill their new responsibilities effectively [6].

ASHA workers have been actively involved in promoting various primary healthcare services, including universal immunization, referral and escort services for institutional deliveries, household toilet construction, and other healthcare interventions, alongside their COVID-19-related activities [7]. They have also taken additional responsibilities like providing essential services such as delivering drinking water and food to individuals in quarantine and isolation and arranging transportation for people when needed. Their pre-existing relationships with the community helped them conduct effective outreach activities. They have continued to make home visits for various purposes, including palliative care, elderly care visits, regular visits for antenatal and newborn care, and home-based childcare. They have put themselves and their families at significant risk of exposure to COVID-19, stigma, violence, and social boycott from their community, with the bare minimum to often no personal protective equipment (PPE) or assured support for priority treatment [8].

The Pradhan Mantri Garib Kalyan Package Insurance Scheme was introduced to provide life insurance coverage for healthcare workers, including ASHAs, who were actively involved in the COVID-19 response. This scheme ensures that in the unfortunate event of death due to COVID-19, the healthcare workers and their families are provided with financial support through life insurance coverage. In addition to the insurance scheme, ASHAs are also eligible for an incentive of Rs. 1,000 per month under the India COVID-19 package [9].

Nationwide, ASHA workers faced various challenges during the COVID-19 pandemic, which impacted their services. These challenges included disruptions in public transport, stigma, restrictions on physical movement, an increased burden of diseases, and the fear of contracting the infection themselves. These factors collectively affected their ability to carry out their duties effectively across India. There have also been accounts of ASHA workers receiving reduced incentives and facing challenges in accessing sufficient protective equipment. These issues were observed in multiple instances throughout the country, highlighting the need for skill development and capacity building for ASHA workers. In addition to monetary rewards, there exists a need for assistance to ASHAs utilizing skill development, collaboration with other community members, and suitable guidance and encouragement, all of which are significantly impacted by the diverse geographical circumstances [10].

These are important lessons to be drawn from the COVID-19 pandemic as far as the functions and challenges of the frontline health workers are concerned. Limited studies have been conducted in Kerala to understand the challenges faced by ASHA workers during the COVID-19 pandemic. Therefore, authorities need to conduct such studies to gain insights into how the pandemic has influenced the roles and responsibilities of ASHA workers in Kerala.

Objectives

The main aims of this study are 1) to explore the impact of the COVID-19 pandemic on the services provided by ASHAs working in a rural Primary Health Center (PHC) in Thrissur district, Kerala, and 2) to understand the measures taken by the ASHAs to fulfill their duties optimally.

## Materials and methods

A qualitative study was conducted in a rural PHC area in Thrissur district during October-November 2021. The study involved 16 ASHAs, a Junior Health Inspector (JHI), and a Junior Public Health Nurse (JPHN) working at the rural PHC. Participants were selected using a purposive sampling method. Before data collection, ethical clearance was obtained from the Institutional Ethics Committee (ECASM-AIMS-2021-402), and consent from the participants was obtained before the study. Two key informant interviews and two focus group discussions (FGDs) were conducted among ASHAs, JHI, and JPHN. Interview guides were developed based on extensive preliminary research. The moderator facilitated the FGDs and the key informant interviews while the note taker recorded detailed notes, captured non-verbal cues and changes in tone, and drew sociograms. The data collection continued until saturation was achieved, and no new themes emerged.

The study employed an inductive thematic analysis approach to explore the impact of the COVID-19 pandemic on services provided by ASHAs, and data triangulation was done. This method was chosen to capture richer data in the ASHAs' own words. Prior to the study, there was limited understanding of the beliefs held by ASHAs, which made a quantitative approach unfeasible. The voice recordings of the interviews were done. The interviews were conducted in Malayalam, which were then transcribed and translated into English. The transcribed notes were coded, and the domains and themes derived from the data analysis were listed accordingly. The domains and themes identified are given in Table 1. 

**Table 1:** Themes and subthemes derived from the analysis ANC, Antenatal Care; ASHA, Accredited Social Health Activist; PPE, personal protective equipment

Themes	Subthemes	Codes
Impact of COVID-19 on work pattern	Intensification of workload	Increased workload during pandemic, shift in focus toward COVID-related tasks
	Temporary cessation of routine duties	Suspension of non-essential tasks during lockdown
	Increased daily working hours	Extension of work hours without adequate rest
	Telephonic ANC follow-up	Adoption of telephonic ANC follow-up methods
COVID-related activities and services	Vaccination	Prioritization and registration of vaccine beneficiaries, addressing vaccine hesitancy and misinformation, challenges in registering beneficiaries on the Cowin portal, managing vaccine distribution logistics
	Medication distribution challenges	Difficulties in obtaining and distributing medicines
	Guidance on precautionary measures	Providing guidance on social distancing and hygiene practices, ensuring compliance with COVID protocols in public spaces
	Additional COVID-related responsibility	New tasks assigned related to COVID management, documentation and reporting of COVID cases
Transportation challenges	Long commuting time	Increased travel time due to lack of public transportation, reliance on private vehicles for transportation
	Financial strain for vehicle purchase	Financial burden of purchasing vehicles, personal expenses incurred for transportation needs
	Ambiguities in patient transfer	Challenges in coordinating patient transfers to treatment centers, resistance from family members toward patient transfers
Safety at work	Challenges in receiving PPE	Limited availability and quality of PPE, financial burden of purchasing PPE
	Family safety concerns	Anxiety about family members' safety
Support systems and gender roles	Family support	Support received from family members, navigating gender roles during the crisis
	Gender role dynamics	Impact of gender roles on work responsibilities, domestic conflicts over financial strain
Community response and social stigma	Initial stigma and misconceptions	Stigmatization of ASHAs as potential carriers, misconceptions about COVID transmission
	Improved health awareness	Increased health-related knowledge among the public, improved health-seeking behavior due to awareness
	ASHA role acknowledgment	Increased empathy and respect towards ASHAs, acceptance of ASHAs as integral members of the community
Duty management strategies	Online training and communication	Online submission of survey details and reports, provision of training modules via online platforms, communication of information and guidelines through WhatsApp
	Shared responsibilities during absences	Redistribution of duties during ASHA absences, ensuring uninterrupted service delivery through teamwork
Financial support and challenges	Government support initiatives	Free COVID testing and monthly COVID incentive
	Financial strain during the lockdown	Job losses and financial difficulties in households, ASHAs becoming sole earners in their families
	Delayed honorarium and financial difficulties	Delays in receiving honorarium payments, financial strain due to interrupted routine services
Need for better working environment	Fixed salary proposals	Need for fixed salary to ensure financial stability
	Increased workforce suggestions	Proposal to increase workforce for better distribution of work
	Addressing medication supply challenges	Suggestions for improving medication supply logistics

Key informants for the study included a JHI and JPHN. Each FGD and interview session commenced with a clear explanation of the study's objectives. During these sessions, data were recorded using a voice recorder. The recorded audio was then translated, transcribed, and systematically coded to identify overarching themes and corresponding subthemes. The primary themes that emerged from the collected evidence encompassed changes in work patterns, social stigmas, safety considerations at work, management of duties, financial implications, shifts in public attitudes, and the requirement for ASHAs.

## Results

The study involved a total of 18 participants, of which 17 were women. The ASHAs who took part in the study had a mean age of 45 ± 4.2 years, and their educational background varied from a minimum of secondary level education to a maximum of higher secondary level education. The work experience of the participating staff members in their current roles ranged from 10 to 13 years. Each ASHA was responsible for covering a population size ranging from 900 to 2,000, including migrant workers. Notably, none of the participants had an alternative source of income (Table 2).

**Table 2:** Sociodemographic profile of the study participants

Category		Frequency (%)
Age (in years)	20-29	1 (5.6)
	30-39	5 (27.8)
	40-49	9 (50)
	≥50	3 (16.6)
Sex	Male	1 (5.6)
	Female	17 (94.4)
Years of experience	≤5	4 (22.3)
	6-10	8 (44.4)
	>10	6 (33.3)
Education	High school	11 (61.1)
	Higher secondary	5 (27.8)
	Degree	2 (11.1)

The themes that emerged from the FGD are as follows.

Impact of COVID-19 on ASHA work pattern

A major finding from this analysis is that the workload for ASHAs had intensified during the pandemic and lockdown period. Before the pandemic, ASHAs followed a standard working schedule that facilitated the completion of their designated responsibilities. They submitted monthly reports to the PHC and participated in review meetings to assess the quality of services rendered.

“We did not have any free time and rest. The awareness classes for pregnant ladies and other beneficiaries were suspended indefinitely. Gatherings and meetings were not allowed.” 

As the initial wave of COVID-19 cases emerged, the entire healthcare system redirected its focus toward containing the spread of the pandemic. Given that ASHAs serve as the primary connection between the community and the healthcare infrastructure, many crucial tasks associated with COVID management were delegated to them. To execute these responsibilities effectively and adhere to social distancing measures, various routine duties, aside from those involving care for pregnant women, palliative care, and tuberculosis cases, were temporarily put on hold during the lockdown. Despite the suspension of their usual tasks, the average daily working hours for ASHAs increased, demanding their constant availability for duties. The follow-up for Antenatal Care (ANC) was done through telephone calls.

A respondent during FGD said:

“We were confused and afraid about the information about COVID and the disease was spreading. We did not have enough time to think. We were deeply involved in the management and care. Every ASHA was in the same situation. People continuously contacted us by phone as they were very tensed.”

COVID-related activities and services

During this period, ASHAs were tasked with taking on several additional responsibilities. These included collating information about travel history, conducting contact tracing for confirmed cases, documenting the health profiles of individuals within households, issuing guidelines for quarantine, overseeing the well-being of quarantined individuals, and distributing medicines, including AYUSH immunity boosters, when necessary. Moreover, ASHAs were engaged in preparing and submitting reports to the medical officer at the PHC. They were also responsible for collecting medications for individuals with hypertension or diabetes from the PHC and facilitating the home delivery of these medications. Obtaining these medicines posed challenges, often requiring them to invest an entire day due to the shortage of pharmacists.

One ASHA responded:

“We had to wait for a long time to get approval signature on the book from the concerned doctors for COVID medicine. If a patient contacted us for medicine in the morning, we could only deliver it in the evening.”

Vaccination

With the initiation of COVID-19 vaccination camps, ASHAs saw a significant increase in their responsibilities. Alongside their existing tasks, they were now tasked with additional duties such as prioritization of beneficiaries for vaccination, gathering necessary information from them, and registering these details in the Cowin portal, the central platform for vaccine registration. Furthermore, ASHAs were entrusted with addressing vaccine hesitancy concerns and addressing misinformation circulating about vaccines. In times of vaccine scarcity, ASHAs encountered difficulties as some members of the public directed their frustration toward them, accusing them of being responsible for the perceived unfair distribution of vaccines. This unfortunate situation sometimes led to arguments between ASHAs and the public.

Some of the ASHAs responded:

“We have not got any rest since the vaccination duty started. We needed to enter details in the Cowin portal a day before vaccination and inform the persons for coming. Some days, the work would get extended till midnight. We were working without food and sleep.”

Public health and social measures

In addition, ASHAs were also tasked with providing guidance to the public regarding precautionary measures, including practicing social distancing, wearing masks, maintaining hand hygiene, and ensuring the safety of senior citizens and young children. They actively visited event venues to ensure adherence to COVID protocols. During the lockdown, numerous students returned to their homes, necessitating ASHAs to gather travel details and offer quarantine recommendations. District medical authorities occasionally requested additional localized information.

ASHAs were also entrusted with delivering medicines to COVID-19 patients. Each ASHA was allocated four to five pulse oximeters from the health center to assist in caring for COVID-19 cases. Due to the limited number of devices, managing a significant number of COVID cases posed challenges. As a solution, they advised some patients to purchase pulse oximeters from medical stores.

“We started follow-up when there were the first few cases, as we maintained a primary, secondary contact list and the list of all people who visited the same place of COVID case visits. During second-wave home, quarantined patients were there, and we needed to supply medicines and pulse oximeters.”

Transport

The commuting time for ASHAs increased due to the lack of public transportation. This forced them to either cover considerable distances on foot or rely on private vehicles to travel from their respective wards to the PHCs. This entailed a significant amount of travel, for which they met from their own pocket. To address this challenge, some ASHAs even had to purchase new vehicles, placing them under financial strain. Moreover, during the initial phase, patients could not be directly transferred to hospitals.

ASHAs were required to relay messages to JHI, who then communicated with medical officers. Only after receiving confirmation from the health department could patients be transferred. In some cases, family members resisted moving patients to treatment centers, leading to unfortunate incidents. The availability of ambulances for nighttime patient transfers was also problematic in certain situations.

Some of the ASHAs’ responses about transportation during FGD were:

“In the absence of public transportation, we had to arrange our transportation facilities to the health center to collect medicines and to supply those to beneficiaries.”

“My husband bought a second-hand scooter and learned driving only to help me in my duty.”

Safety at work

ASHAs faced unsafe working conditions due to insufficient protective gear and a lack of acknowledgment as government health workers. The availability of protective masks and equipment was limited, prompting ASHAs to buy them with their funds. Many healthcare workers, including JPHN and JHI, used disposable masks, and some reused them, even though they received enough protective gear at the PHC.

A few received minimal protective equipment, such as a small bottle of sanitizer and a single gown as PPE, which quickly deteriorated.

The response from one ASHA about safety was:

“We were distributing medicines to patients without even caring for our safety. We bought gloves and masks with our own money. During this time, many ASHAs were infected with COVID-19.”

Support systems and gender roles

Given the nature of their work, ASHAs faced a higher risk of contracting the infection. Respondents expressed concern not only for their safety but also for the potential dangers posed to their families. Although most ASHAs received good support from their families, some were anxious about navigating gender roles during the crisis. Some reported domestic disharmonies over the financial hardship caused by the pandemic.

Community response and social stigma

In the initial phase of the pandemic, neighbors considered ASHAs as potential carriers upon their return from work. Over time, as people became more aware, they began to show empathy and respect towards ASHAs. To safeguard their household members, ASHAs predominantly relied on practices such as washing their hands, bathing, and promptly cleaning their uniforms upon returning from work.

During the initial stages of COVID-19 management, many ASHAs encountered resistance and conflicts from residents who were hesitant to comply with the implemented restrictions. As the awareness about the situation increased over time, ASHAs began receiving recognition and appreciation from the public.

One of the responses related to the change in public attitude was:

“COVID changed people a lot, and now, they are contacting us regularly to share their health updates.”

This shift in public attitude was due to the assistance provided by ASHAs during challenging times. They extended aid by supplying essential items to isolated households, ensuring water access for homes without connections, and supplying food to those in need. As a result of these actions, ASHAs gradually earned the esteem of the community, with many people now considering them as part of their own families. This growing sense of belonging within society serves as a motivating factor for ASHAs. Support came from various places as well. ASHAs received support from their superiors and ward members, who assisted them through various challenges.

An ASHA, during a discussion about social stigma, said:

“One day, a child from a neighborhood came toward me, but her parents warned her not to touch me as I was on COVID duty. I was very sad after this. We even did not enter the patient’s house; we just kept the medicine on a chair outside houses or on the flower pot.”

Duty management strategies

ASHAs were asked to submit the survey details and other information online at any time, so they had to be ready with reports regardless of the time of the day. Review meetings were not conducted in the health center, but supervisors coordinated the duties. The training modules were provided to them via online platforms, and information and guidelines changes were updated by health inspectors without delay. All the ASHAs had smartphones, and they received enough guidelines and information over WhatsApp on a timely basis. They maintained a WhatsApp group for the sole purpose of COVID control, and it was easy for them to communicate with peers and authorities. They raised awareness about telemedicine among the people and encouraged them to contact telemedicine in times of emergencies. When one ASHA became positive for COVID-19 or when they were in quarantine following contact with a case, the duties were shared by other ASHAs. Thus, they ensured the delivery of unbroken services.

A lady reported on duty management strategies:

 *“We did good teamwork to lower the rate of spread and deaths in the initial stage of COVID and received awareness classes regarding guidelines and protocols that we have to follow at the time of contact with the COVID patients, and we used to receive a daily update via PHC WhatsApp group.”*

Financial support and challenges

The government has extended financial support by offering free COVID testing and providing a monthly COVID incentive of Rs. 1,000. Due to the interruption of routine services, the incentives earned were less compared to the previous months. During the lockdown, some respondents reported that other earning members of their households lost their jobs and did not receive wages for March and April. Consequently, ASHAs became the sole earners in their families. They also experienced delays in receiving the honorarium during certain months, which caused a lot of financial difficulties.

Need for better working environment

The ASHAs engaged in discussions to tackle the barriers they had identified and to boost their motivation for work. One main suggestion was to provide them with a fixed salary, considering their status as incentive-based work. Some suggested increasing the workforce to ensure a more efficient division of labor, thereby enhancing effectiveness. They were also engaged in the supply of medicines at the sub-center level, which involved extensive travel, which was also addressed as a common challenge.

“During COVID spread, we were on duty, leaving our household things behind. Noncommunicable disease clinics will be there on some days with 20 patients. We had to buy the medicines for them along with the supply of medicine for COVID-19 patients. It might take a whole day of waiting because the staff had to supply medicine to their patients first. By then, people started calling us and complaining about being late.”

Supervisor's role

The interviews conducted with the JHI and JPHN gathered valuable insights into the supervisor's role in supporting ASHA workers. They played a crucial role in coordinating duties, providing training, ensuring timely communication of COVID-19 protocols, and helping to resolve challenges like transportation and supply chain issues. They also helped ASHAs by providing advice when needed.

## Discussion

The ASHAs play a crucial role in our healthcare system and are an essential component of community processes. The findings of this study emphasize the impacts of COVID-19 on the services provided by ASHA. The pandemic intensified the work of ASHA and shifted the priorities to focus attention on COVID-19 containment and management of cases. During this time, ASHAs encountered numerous obstacles in carrying out their responsibilities, such as public attitudes and lockdown protocols. The main factors driving their work performance were altruism, community esteem, and acknowledgment. Similar findings were reported in a qualitative study by Singaraju et al., which was conducted in six states of India - Andhra Pradesh, Assam, Haryana, Tamil Nadu, Telangana, and West Bengal in 2020 [11]. According to the Women in Global Health India chapter, the issues faced by ASHAs during the pandemic were increased burden of work, inadequate training to undertake the new roles, denial of adequate safety from violence and infections, their rightful demands of recognition and respect from government as well as community, and, most importantly, the lack of appropriate and timely remuneration [8]. Increased workload and financial constraints were also observed in a study done by Devarajappa et al. in Karnataka [12]. Similar issues among ASHA workers, such as inadequate training, lack of safety gear, and low remuneration, were also observed in our study. 

According to a study done by Bhaumik et al., community health workers were involved in COVID-19-related tasks, such as raising awareness about the disease, implementing precautionary measures, contact tracing, coordinating patient care, providing support, and addressing stigma at the community level in addition to routine work [13]. Similar findings were observed in our study, and these additional responsibilities disrupted their routine work.

In a study done by Reji et al. in Poonthura Village in Kerala, fear and anxiety were common among ASHA workers when discussing their personal lives during COVID-19. They had the fear of getting sick and spreading the infection to their family members including children [14]. Anxiety about family members' safety was also noted among our study participants. In a study on the Impact of the Pandemic on ASHA in India by Singaraju et al., challenges experienced during COVID time were an intensification of workload, additional responsibility, and increased commuting time. These findings closely resemble the results of our study [11].

Many ASHAs received significant support from their families, enabling them to fulfill their duties effectively. Similar results were reported in other studies, such as a mixed-method study by Chopra P et al., which revealed no negative changes in the attitudes of family members across various parts of India [15]. The majority of ASHAs had received basic informative sessions and training, and they were satisfied with the way it was provided. However, in a study done by Chowdhury et al. in Tripura, a relatively small proportion of ASHA had adequate knowledge about COVID-19, and they felt that dissemination of information and proper training are crucial during emergencies like the COVID-19 pandemic [16].

Limitations

More number of interviews and FGDs would have been conducted for better generalizability of results. The study was conducted around a single PHC area and stakeholder perspectives were not captured.

## Conclusions

This study reveals that COVID-19 has affected the services of ASHA in many ways. The COVID-19 pandemic led to a significant shift in the responsibilities of ASHAs, with their role as "healthcare providers and facilitators" taking precedence over all other tasks. This shift resulted in an increased workload, experiences of stress and anxiety, and a backlog of routine tasks. However, ASHAs received widespread appreciation for their frontline activities during this challenging time.

There were many facilitators, such as social support, teamwork, and self-satisfaction in services. There were some barriers present in their uninterrupted services, such as insufficient and irregular remuneration, lack of recognition, problems with transportation, safety issues, and out-of-pocket expenses. ASHAs managed the adverse situations by taking proper measures, and they were very keen and sincere in executing their duties. However, more research is required to understand the impacts of the COVID-19 pandemic on ASHA’s duties in diverse settings across India.
